# A comprehensive analysis of the history of DFT based on the bibliometric method RPYS

**DOI:** 10.1186/s13321-019-0395-y

**Published:** 2019-11-21

**Authors:** Robin Haunschild, Andreas Barth, Bernie French

**Affiliations:** 1 Max Planck Institute for Solid State Research, Heisenbergstraße 1, 70569 Stuttgart, Germany; 2 FIZ Karlsruhe - Leibniz Institute for Information Infrastructure, Hermann-von-Helmholtz-Platz 1, 76344 Eggenstein-Leopoldshafen, Germany; 3 CAS Innovation LAB, CAS (Chemical Abstracts Service), A Division of the American Chemical Society, 2540 Olentangy River Road, Columbus, OH 43202-1505 USA

**Keywords:** DFT, Density functional theory, CRExplorer, Reference publication year spectroscopy, RPYS, Historical roots, Seminal papers

## Abstract

This bibliometric study aims at providing a comprehensive analysis of the history of density functional theory (DFT) from a perspective of chemistry by using reference publication year spectroscopy (RPYS). 114,138 publications with their 4,412,152 non-distinct cited references are analyzed. The RPYS analysis revealed three different groups of seminal papers which researchers in DFT have drawn from: (i) some long-known experimental studies from the 19th century about physical and chemical phenomena were referenced rather frequently in contemporary DFT publications. (ii) Fundamental quantum-chemical papers from the time period 1900–1950 which predate DFT form another group of seminal papers. (iii) Finally, various very frequently employed DFT approximations, basis sets, and other techniques (e.g., implicit descriptions of solvents) constitute another group of seminal papers. The earliest cited reference we found was published in 1806. The references to papers published in the 19th century mainly served the purpose of referring to long-known physical and chemical phenomena which were used to test if DFT approximations deliver correct results (e.g., Van der Waals interactions). The foundational papers of DFT by Hohenberg and Kohn as well as Kohn and Sham do not seem to be affected by obliteration by incorporation as they appear as pronounced peaks in our RPYS analysis. Since the 1990s, only very few pronounced peaks occur as most years were referenced nearly equally often. Exceptions are 1993 and 1996 due to seminal papers by Axel Becke, John P. Perdew and co-workers, and Georg Kresse and co-workers.
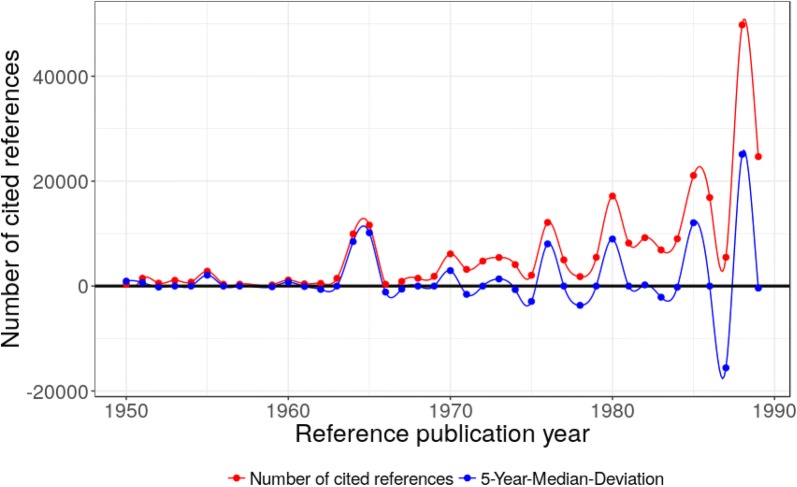

## Introduction

The terms bibliometrics or scientometrics (in a broader sense) are often used synonymously and can be characterized as the discipline that provides a quantitative [1, 2] overview about science. The most basic quantities used in bibliometrics are publication and citation counts. They are used to construct bibliometric indicators for research evaluation purposes. The perspective of research evaluation looks forward in time from the individual publication and the corresponding citing papers are counted. In this study, we turn the perspective backwards from the individual publication and analyze the cited references (i.e., the number of times a specific reference is included in the reference lists). One approach of such an analysis is called reference publication year spectroscopy (RPYS). This bibliometric method can be used to locate seminal papers which are cited most frequently in a certain publication set [3]. The question about seminal papers in a given field can be answered by researchers in the field only in a subjective way. RPYS, however, can answer this question in an objective way by asking all researchers in the field (via the cited references in their publications) with subsequent quantitative analysis. Therefore, RPYS results often provide a different perspective or complement the individual expert’s perspective on the field. For example, RPYS analyses have been performed to discover the historical roots of individuals [4], publications in a journal [5, 6], or research fields [7]. Very large publication sets can be analyzed by sampling methods implemented in the CRExplorer [8]. An overview of further studies based on RPYS can be found in Marx and Bornmann [9]. In this paper, we were able to use a very large data set as the basis for our analysis to cover the complete research field of density functional theory (DFT) and its applications from a chemical perspective.

Kohn–Sham DFT [10] has become one of the most important methods to solve the Schrödinger [11, 12] and Dirac [13–15] equations approximately. Besides the foundational theorems by Hohenberg and Kohn [16], DFT stands on many other pillars. Thomas [17] and Fermi [18] proposed the very first density functional approximation without mentioning the term. The simplifications of the Hartree–Fock [19, 20] method by Slater [21] has enabled practical DFT calculations. Kohn–Sham DFT calculates the energy of a non-interacting reference system. Exchange and correlation functionals are used to approximate the difference to the real system. The simplest exchange and correlation functionals depend only on the electron density itself [22]. The development of exchange and correlation functionals that also included the gradient (GGA functionals) [23–26] and second derivatives (meta-GGA functionals) [27–29] of the electron density permitted more accurate calculations. GGA and meta-GGA calculations provide higher accuracy in general at negligible additional computational expense. DFT calculations became even more accurate and attractive with the development of hybrid functionals [30–35]. However, hybrid functionals increased also the computational expense significantly. Admixture of a global fraction of Hartree–Fock exchange yielded higher accuracy for atoms and molecules, and applicability of hybrid functionals to solids and surfaces was enabled by range-separation with a screened Coulomb potential [36–38]. Long-range-corrected hybrid functionals provided more accurate calculations of reaction barrier heights [39–41]. The accuracy of DFT calculations was increased even more by the admixture of a variable fraction of Hartree–Fock exchange, as done by local hybrid functionals [42–48]. Combination of the concepts of local hybrids and range-separated hybrids [49–51] increased the accuracy even further. In addition, correlation from wave function methods (MP2 [52, 53], RPA [54–57], coupled-cluster [58–60]) was also admixed with the original correlation functional [61, 62]. Time-dependent DFT, based on the work of Runge and Gross [63], has become a well-established methodology for treating electronically excited states.

The history of quantum chemistry for DFT and ab initio quantum chemistry and many-body perturbation theory (MBPT) has been discussed by Kutzelnigg [64, 65]. Although such a qualitative review on DFT and its historical roots from the perspective of an individual researcher is very helpful, a quantitative overview based on large publication sets can only be obtained using bibliometric methods. There is considerable interest in the evolution of the annual publication volume in the field of DFT [66, 67]. Recently, Haunschild et al. [68] provided a bibliometric overview about DFT publications. We intend to extend this bibliometric effort in this study by presenting a quantitative overview about the historical roots and seminal publications of DFT for the time period from 1800 until 2012 using RPYS.

## Methodology

Our analysis is based on the application of the search and retrieval functions of STN^®^ to the Chemical Abstracts Plus literature database (CAplus^SM^) provided by CAS (Chemical Abstracts Service), a division of the American Chemical Society (ACS). The CAplus database covers scientific publications and patents related to chemistry since around 1900 (including the references cited therein since the publication year 1996).

The CAplus publication records contain index terms (IT) which have been carefully selected and assigned by the database producer (CAS). We searched for the terms “DFT”, “density functional theory”, “d functional theory”, and “TDDFT” in the IT fields of the CAplus database. Occurrences of “TD-DFT” and “time-dependent density functional theory” are also found by our search terms. The search term “d functional theory” is not used by scientists using DFT but it is used by CAS indexers. In total, we found 114,138 documents published before the end of the year 2014 (at the date of searching the year 2015 was not completely covered by the database). Throughout this paper, we will refer to this set of 114,138 documents as “DFT publications”. Although indexing takes some time, we can expect that the publication years until 2014 are nearly complete. Searching in the IT field of CAplus has the advantage that only documents are retrieved where DFT plays a major role (e.g.: where DFT methods are employed or developed). Documents in which DFT is mentioned along the way in the abstract are not retrieved. This reduces the citation count in our study in comparison with citation counts from other databases.

We analyzed the DFT publications with respect to seminal papers and historical roots on which the DFT publications are based. Such seminal papers can be located using a bibliometric method called “reference publication year spectroscopy” (RPYS) [3] in combination with a recently developed tool named CRExplorer (http://www.crexplorer.net) [69]. The analysis of the publication years of the references cited by all the papers in a specific research field shows that (especially earlier) publication years are not equally represented. Some years occur particularly frequently among the references showing up as pronounced peaks in the distribution of the reference publication years (i.e., the RPYS spectrogram). In most cases, the peaks are based on single publications, which are highly cited compared to other early publications. It is assumed that the highly cited papers are of specific significance to the research field in question (here: DFT).

In a first step, the publication set is imported into the CRExplorer and all cited references are extracted. In a second step, equivalent references are clustered and merged. References occurring less often than a certain threshold (see below) are removed to reduce the background noise and to sharpen the resulting spectrogram. In the third and final step, the reference publication years are analyzed for frequently cited publications. Older RPYs require a slightly different methodology, i.e., a lower threshold of the minimum number of cited references because the scale for the number of cited references (NCR, i.e., count of publications which cited a specific reference) differs significantly across different periods of time. The 114,138 DFT publications contain 4,412,152 non-distinct cited references. Handling (clustering, merging, and analysis) of such a large number of cited references is non-trivial. Therefore, we divided our analysis into four different time periods: (1) 1800–1899, (2) 1900–1949, (3) 1950–1989, and (4) 1990–2012. Between 1800 and 1899 the maximum peak height is 125, between 1900 and 1949 it is over 3000, between 1950 and 1989 it is over 50,000, and finally for the last period it has raised to around 60,000.

The threshold for references to be removed for the first two time periods (1800–1899 and 1900–1949) is 10. For the third time period (1950–1989), we used the threshold of 100 consistent with our earlier study in this time period [68]. The last time period (1990–2012) contained by far the most cited references. Therefore, we applied the final threshold of a minimum of 100 also to this time period after clustering and merging of reference variants.

Reference variants can occur in high numbers. As an example, we point out the number of reference variants to the very popular computational program Gaussian: (1) to the 2003 version of the program package and (2) to all different program versions. We found 2035 different reference variants amounting to 18,397 cited references of the 2003 version. This would put this version of Gaussian between CR69 and CR72 (see “Appendix”). More than 4000 reference variants could be identified for any version which amounts to 43,736 cited references. This makes the program package Gaussian referenced more often than any other publication in our set. However, as we are interested in scientific publications, we removed references to program packages (i.e., Gaussian and SHELX).

Some typos in the publication year or permutations of publication year with page number were spotted. For example, we found a reference to “KRESSE G, 1758, PHYS REV B, V59, P1999” and the correct reference is “KRESSE G, 1999, PHYS REV B, V59, P1758” (CR79). Another example is “PERDEW J, 1092, PHYS REV B, V46, P6671”. The correct cited reference is “PERDEW J, 1992, PHYS REV B, V46, P6671” (CR68). However, these errors were not corrected because they occurred rather seldom.

## Results

In a previous paper, we have briefly discussed the history of DFT [68] for the period from 1950 to 1989. In this paper, we analyze the history of DFT for the time period between 1800 and 2012. Since DFT was founded in 1964, when the famous Hohenberg–Kohn theorems [16] were published, it is obvious that our dataset must contain many references to important precursor papers which are indirectly related to DFT. Since the number of peak papers is rather large (n = 85) we have decided to focus our analysis on the most important papers only and provide the complete list of peak papers in “Appendix”.

In our study of the history of DFT, we find that the 19th century is characterized by studies of special phenomena in physics, preparations and reactions of special chemical compounds, as well as some theoretical precursors to DFT. The first half of the 20th century is characterized by the discovery of quantum mechanics and its applications to atomic and molecular structures and their related physical and chemical phenomena. In the light of DFT, this period is dominated by the paper from Møller and Plesset [70] on perturbation theory. In the period from 1950 to 1989, DFT was founded by Hohenberg and Kohn [16], and Kohn and Sham [10]. In the aftermath, several approximations have been developed and applied to new and old problems in chemistry and physics. In the final period from 1990 to 2012, new approximations were assessed and the results demonstrate the success of DFT, especially for the calculation of larger molecules.

### Time period 1800–1899

**Fig. 1:**
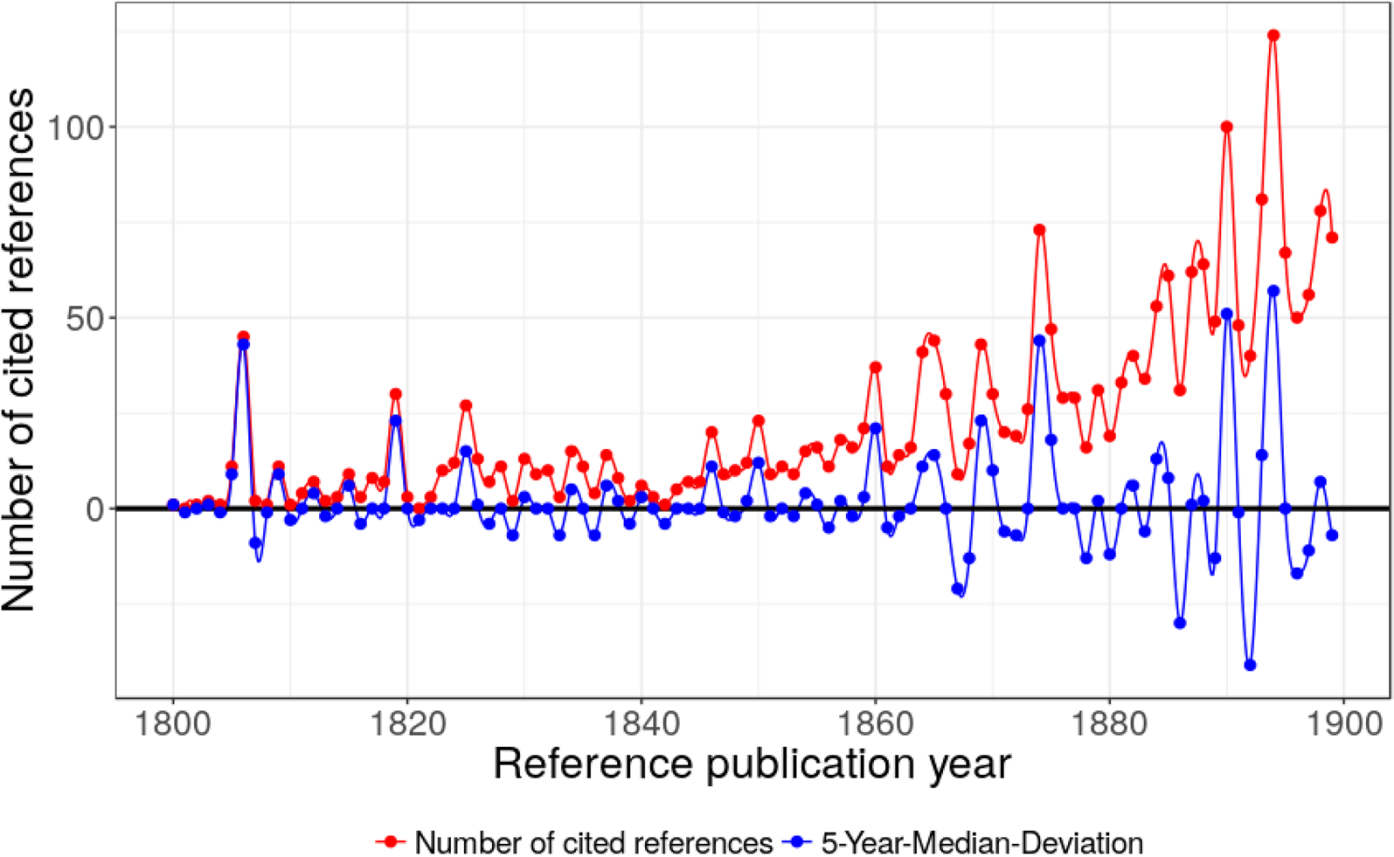
Annual distribution of the references cited in DFT publications across their reference publication years within the time period 1800–1899. The major peak positions are at 1806, 1819, 1825, 1846, 1850, 1860, 1865, 1869, 1874, 1885, 1890, 1894, and 1898

In the period from 1800 to 1899, we find a spectrogram with several rather small peaks (see Fig. 1). The red points and curve in Fig. 1 show the number of cited references (NCR) in each reference publication year (RPY) while the blue points and curve show the 5-year median (x − 2, x − 1, x, x + 1, and x + 2) deviation from the NCR in the specific RPY. This color scheme is also used for the other RPYS figures in this paper.

As can be seen in Table 1 (in “Appendix”), the set of peak papers can be roughly divided into papers with a focus on physics, physical chemistry, and classical organic chemistry. The set of physics and physical chemical papers comprises the Grotthuss mechanism for proton transfer in water (CR1, 1806), the Petit-Dulong rule to determine molar heat capacities (CR2, 1819), Michael Faraday’s work on the nature of light in magnetic fields (CR4, 1846), van der Waals theory on capillarity (CR13, 1894), and the combining rule of Marcellin Berthelot for the calculation of the Lennard-Jones potential (CR15, 1898).

Chemical discoveries include the identification and preparation of several new compounds: benzene by Michael Faraday (CR3, 1825) whose corresponding structural formula was first proposed by August Kekulé (CR7, 1865), the preparation of α-amino acids from aldehydes/ketones, ammonia, and cyanide by Adolph Strecker (CR5, 1850), the synthesis of salicylic acid by Hermann Kolbe (CR6, 1860), the Glaser coupling of two terminal alkines (CR8, 1869), the synthesis of long carbon chains by Adolf von Baeyer (CR10, 1885), the rearrangement of an acyl azide to an isocyanate by Theodor Curtius (CR12, 1890), the discovery of nickel carbonyl and the class of metal carbonyls by Ludwig Mond (CR11, 1890), and Henry John Horstman Fenton invented a reagent which can be used to destroy certain organic compounds (Fenton’s reagents) (CR14, 1894). In addition, stereochemistry was invented by Jacobus Henricus van’t Hoff (CR9, 1874). All these discoveries and inventions have been re-examined in the light of quantum mechanics by applying various approximations of DFT, mainly to test certain density functionals regarding well-known phenomena.

### Time period 1900–1949

**Fig. 2:**
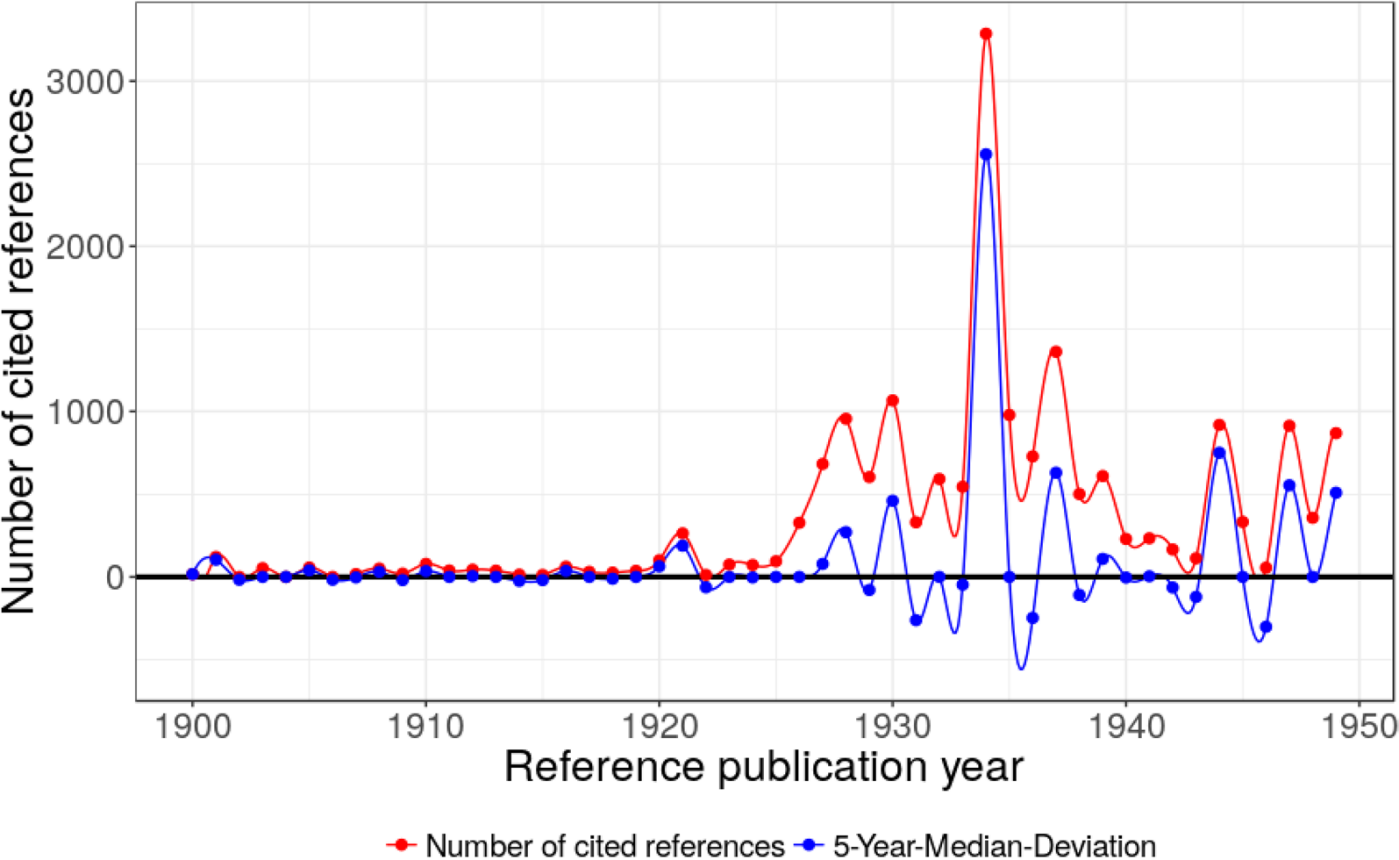
Annual distribution of the references cited in DFT publications across their reference publication years within the time period 1900–1949. The major peak positions are at 1901, 1921, 1928, 1930, 1934, 1937, 1944, and 1947

The scientific progress in the first half of the 20th century was largely dominated by the development of the fundamental theories in physics. Quantum mechanics was discovered, and the concepts were applied to atoms and molecules, new analytical tools were invented which enabled scientists to study the atomic and sub-atomic world, and an initial understanding of the nature of the chemical bond was gained. A part of this history is reflected in Fig. 2 (and Table 2 in “Appendix”) from the perspective of DFT.

Although Table 2 (in “Appendix”) contains 35 frequently cited papers we will concentrate here on the most significant peak papers. Aside from the journal publications, we also find three important books: a textbook on crystal physics by Woldemar Voigt (1850–1919) (CR28, 1928, in German), an early introduction to quantum chemistry by Hans Hellmann (1903–1938) (CR43, 1937, in German) and a handbook on Infrared and Raman Spectra of Polyatomic Molecules by Gerhard Herzberg (1904–1999) (CR49, 1945).

In 1901, George Wulff published a paper on the growth rate and the dissolution of crystal surfaces. He also defined the so-called Wulff construction, a method which allows the determination of the equilibrium shape of a droplet or a crystal of a fixed volume (CR16, 1901). Paul Ewald calculated optical and electrostatic grid potentials in which he proposed a method to analyze dipole fields based on the theta function (CR22, 1921). In the same year, Lars Vegard published a paper on the constitution of mixed crystals and the space occupied by atoms (CR23, 1921). In 1928, only 1 year after the publication of the Schrödinger equation, Enrico Fermi calculated atomic properties using a statistical approach where he treated the electrons as a perfect gas with complete degeneration (CR27, 1928). In the same year, Douglas Hartree published the so-called Hartree equations for the calculation of many-electron systems in a self-consistent field (CR29, 1928). These equations were generalized by Vladimir Fock to include exchange phenomena between two electrons (CR33, 1930) and they are known today as the Hartree–Fock equations. The Hartree–Fock method is an integral part of many quantum chemical calculations including DFT applications [10]. Paul Dirac published a note on the exchange phenomena in the Thomas atom (CR32, 1930) and Carl Eckart proposed a theory to explain the penetration of a potential barrier by electrons (CR34, 1930).

Tjalling Koopmans proposed an approximation for the calculation of ionization energies which is known today as Koopmans’ Theorem (CR38, 1934). In the same year, Christian Møller and Milton S. Plesset proposed a perturbative treatment of many-electron systems (CR37, 1934). This theory is often used as a reference method to benchmark new functionals for larger molecules. Furthermore, hybrid correlation functionals mix correlation from DFT with correlation from wave function methods, e.g. MP2 [70], RPA [52, 54, 61]. The second-order treatment (MP2, if used on top of the Hartree–Fock method) has been employed most often due to the good compromise between increased accuracy and computational demand. This approach is a central pillar of DFT approximations which combine wave function correlation with density functional correlation, and hence it is not surprising that this publication is cited very frequently, and the corresponding peak completely dominates the time period 1900–1949.

Fritz London developed a theory for the description of interatomic currents in aromatic compounds (CR42, 1937). In the same year, Hermann Arthur Jahn and Edward Teller published a new theorem which was later called the Jahn–Teller effect (CR44). This effect describes the spontaneous symmetry breaking in molecules and solids. Francis Dominic Murnaghan developed an equation of state which describes the relationship between the volume and the pressure of a body (CR48, 1944). Francis Birch formulated the so-called Birch-Murnaghan isothermal equation of state based on Murnaghan’s ideas (CR50, 1947).

### Time period 1950–1989

During this time period, we find several distinguished peaks, but the spectrogram is dominated by a single peak in 1988 (see Table 3 in “Appendix”). This one is actually caused by two very highly cited papers from Lee et al. [26] and from Becke [25]. In addition, there are several papers dealing with extensions of the Hartree–Fock equations as well as applications of molecular orbital (MO) theory. More important are the papers by Hohenberg and Kohn [16], and Kohn and Sham [10] with their fundamental work on DFT.

John C. Slater proposed an approximation to the Hartree–Fock exchange potential (CR51, 1951), and Clemens C. J. Roothaan (CR52, 1951) developed the concept of molecular orbitals as a linear combination of atomic orbitals (LCAO). LCAO was initially applied to Hartree–Fock theory but it is used in virtually every widespread program package for post-Hartree–Fock and DFT calculations. A few years later Robert S. Mulliken (CR53, 1955) proposed an electronic population analysis based on Roothaan’s LCAO method. Using this methodology, it became possible to calculate partial charges and dipole moments.

The foundational publications for modern DFT by Pierre Hohenberg and Walter Kohn (CR54, 1964) and Walter Kohn and L. J. Sham (CR55, 1965) were published in 1964 and 1965. Hohenberg and Kohn [16] postulated and proved the Hohenberg–Kohn theorems which build the foundation of DFT. Kohn and Sham [10] provided a practical methodology (Kohn–Sham equations) based on the ideas behind the Hartree–Fock equations to apply DFT to molecules and solids.

S. Francis Boys and Fernando Bernardi developed a new direct difference method for the computation of molecular interaction energies with reduced errors (CR56, 1970). Warren J. Hehre, Robert Ditchfield, and John A. Pople (CR57, 1972) presented new basis sets for the LCAO method. The 6-31G basis set, which became very popular, is among those basis sets presented in this cited reference. The relevance of polarization functions was pointed out by Puthugraman C. Hariharan and John A. Pople (CR58, 1973), and the popular 6-31G* and 6-31G** basis sets were introduced. Jan Evert Baerends, Donald E. Ellis, and Piet Ros (CR59, 1973) presented a computational Hartree–Fock scheme using Slater’s approximation and Roothaan’s LCAO ansatz. CR60 (1976) is the only cited reference in Table 3 (in “Appendix”) specifically concerned with the solid state. The authors propose a method for generating sets of special points in the Brillouin zone. This method provides a more efficient algorithm to integrate periodic functions of the wave vector in solid state calculations.

Between 1980 and 1988 we find four publications of new density functional approximations or improvements to existing ones (CR61, CR64-CR66) and two publications of effective core potentials (CR62, CR63). Seymour H. Vosko, L. Wilk, and Marwan Nusair proposed popular local correlation functionals (CR61, 1980). P. Jeffrey Hay and Willard R. Wadt proposed effective core potentials for the atoms K-Au and Sc–Hg which enable a cost-effective implicit treatment of inner-shell electrons for heavier elements (CR62, CR63, both 1985). John P. Perdew (CR64, 1986) proposed a gradient correction to an earlier local correlation functional developed by John P. Perdew and Alex Zunger. The references to the publications by Chengteh Lee, Weitao Yang, and Robert G. Parr on the *development of the Colle*-*Salvetti correlation*-*energy formula into a functional of the electron density* (NCR = 23,953, CR65) and by Axel Becke on *density*-*functional exchange*-*energy approximation with correct asymptotic behavior* (NCR = 14,150, CR66) completely dominate the peak in 1988. Lee et al. [26] proposed a correlation functional (LYP) and Becke [25] proposed an exchange functional (B, also known as Becke88). Both functionals were combined in the highly popular functionals BLYP, B3LYP (see below), and many others. Those functionals were implemented in very popular program packages (e.g., Gaussian) and thereby made available to the computational chemistry community. The extraordinary many citations to these publications are easily explained because they are cited each time a functional containing Becke88 exchange and/or LYP correlation is used in a study. Although there is a considerable number of functionals containing Becke88 exchange and/or LYP correlation, the very high NCR values are due to the popularity of some of these functionals, especially BLYP and B3LYP.

### Time period 1990–2012

In this time period, we find several highly and very highly cited papers and sometimes several peak papers have been published in a single year (see Table 4 in “Appendix”). In this period, several sophisticated approximations to the exchange potential of the DFT equations have been developed. Although there are peaks for the years 1992/1993/1994, 1996, and 1998/1999, there are two dominating peaks in 1993 and 1996 (see Fig. 4). The peak in 1993 is caused by a paper from Becke [30] entitled *Density*-*functional thermochemistry. III. The role of exact exchange* with 25,970 cited references. In 1996, we find three major papers, one from Perdew et al. [24] and two from Kresse and Furthmüller [71, 72] with a total of 29,522 cited references. For both peaks this is roughly half of all cited references in these years.

John P. Perdew and Yue Wang developed an analytic representation of the correlation energy for a uniform electron gas (CR67, 1992) and they demonstrated the accuracy in several numerical tests (CR68, 1992). A year later, Axel Becke proposed the family of hybrid functionals where DFT exchange is mixed with Hartree–Fock exchange (CR69, 1993). The first member of this new family of functionals has become known as B3PW91 (abbreviation used for: Becke, three parameters, Perdew–Wang-91) which performed significantly better than previous functionals with gradient corrections only. Due to the high accuracy of this approximation it became very popular and the paper turned out to become the most highly cited paper in this time period. Peter E. Blöchl (CR70, 1994) developed the projector augmented-wave method (PAW) which is a generalization of both the pseudopotential and the linear augmented-plane-wave (LAPW) method. In the same year, Philip J. Stephens, Frank J. Devlin, Cary F. Chabalowski, and Michael J. Frisch (CR71, 1994) proposed to use LYP correlation instead of PW91 correlation in the B3PW91 functional without adjustment of the three parameters. This led to the highly popular B3LYP functional. Furthermore, they applied several approximations to calculate vibrational transition spectra of the chiral 4-methyl-2-oxetanone and showed that B3LYP is in excellent agreement with experiment. Therefore, this publication is usually cited when B3LYP is used in studies. Later, Reiher et al. [73] refitted the parameters and named the resulting functional B3LYP*. Although Reiher et al. [73] is none of our peak papers, it does occur in our RPYS analysis with 309 cited references. The original B3LYP functional stayed far more popular than the refitted version.

John P. Perdew, Kieron Burke, and Matthias Ernzerhof published a derivation of a generalized gradient approximation (GGA) for the electron correlation and exchange energy (1996, CR72) which is known as PBE, named after the authors of the paper. PBE is a very popular and computationally rather inexpensive density functional (exchange and correlation) for molecules and solids. In the same year Georg Kresse and Jürgen Furthmüller (CR73, CR74, both 1996) presented an efficient scheme for calculating the Kohn–Sham ground state of metallic systems using pseudopotentials and a plane-wave basis set. The algorithms were implemented within a program package called VASP (Vienna ab initio simulation package). The peak in 1998 is based on four papers (CR75–CR78). Vincenzo Barone and Maurizio Cossi (CR75) performed quantum-chemical calculations of molecular energies and energy gradients in solution by a conductor solvent model. This paper also describes the COSMO (conductor-like screening solvation model) implementation in Gaussian. It is cited in studies which use the Gaussian implementation of COSMO for modelling solvated molecules. Mark E. Casida, Christine Jamorski, Kim C. Casida, and Dennis R. Salahub (CR76) and R. E. Stratmann, Gustavo E. Scuseria, and Michael J. Frisch (CR77) applied time-dependent density functional theory (TDDFT) to the calculation of excitation energies. Carlo Adamo and Vincenzo Barone (CR78) constructed exchange functionals with an improved long-range behavior.

Georg Kresse and Daniël P. Joubert (CR79, 1999) showed the formal relationship between ultra soft pseudopotentials and the projector augmented wave (PAW) method and provided critical tests of both methods calculating both small molecules as well as bulk systems. Carlo Adamo and Vincenzo Barone (CR80, 1999) constructed the popular hybrid functional PBE0. The parameter to determine the amount of Hartree–Fock exchange is derived from an argument from perturbation theory. Therefore, PBE0 is also considered to be a non-empirical hybrid functional.

In 2001, G. te Velde and co-workers published a detailed review of the Amsterdam Density Functional (ADF) program (CR81). Jacopo Tomasi, Benedetta Mennucci, and Roberto Cammi published a review about quantum mechanical continuum solvation models for the implicit description of solvents and solvation effects (CR82, 2005). Florian Weigend and Reinhart Ahlrichs (CR83, 2005) provided basis sets of different sizes for most elements of the periodic table. These basis sets (also so called Ahlrichs or Karlsruhe basis sets) are an integral part of the program package Turbomole, and they are also often used by users of other program packages. The influence of different basis sets on the calculation of atomic and molecular properties is also analyzed in CR83. In 2006, Stefan Grimme augmented Becke’s functional B97 with empirical, damped, and atom-pairwise dispersion corrections (CR84) which performed especially well for long range van der Waals interactions. Yan Zhao and Donald G. Truhlar developed a set of highly parametrized functionals (CR85, 2008) which they applied successfully to organometallic and inorganometallic compounds.

## Discussion

**Fig. 3:**
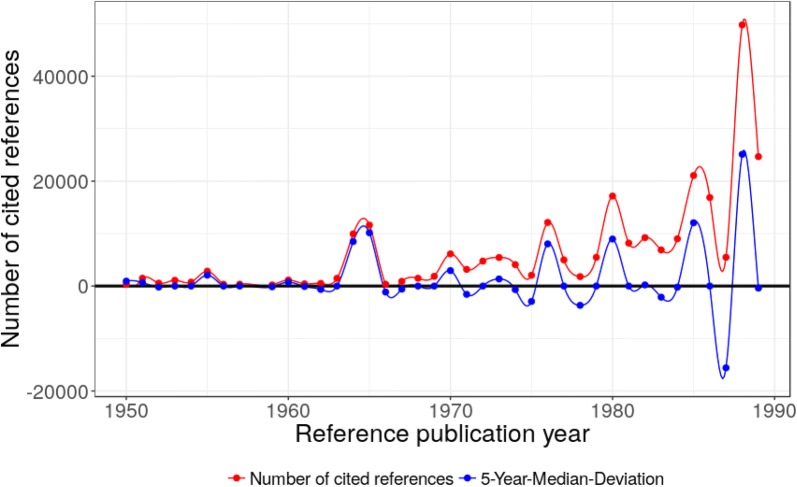
Annual distribution of the references cited in DFT publications across their reference publication years within the time period 1950–1989. Only references with a minimum reference count of 100 are considered here. The major peak positions are at 1955, 1964/1965, 1976, 1980, 1985/1986, and 1988

**Fig. 4:**
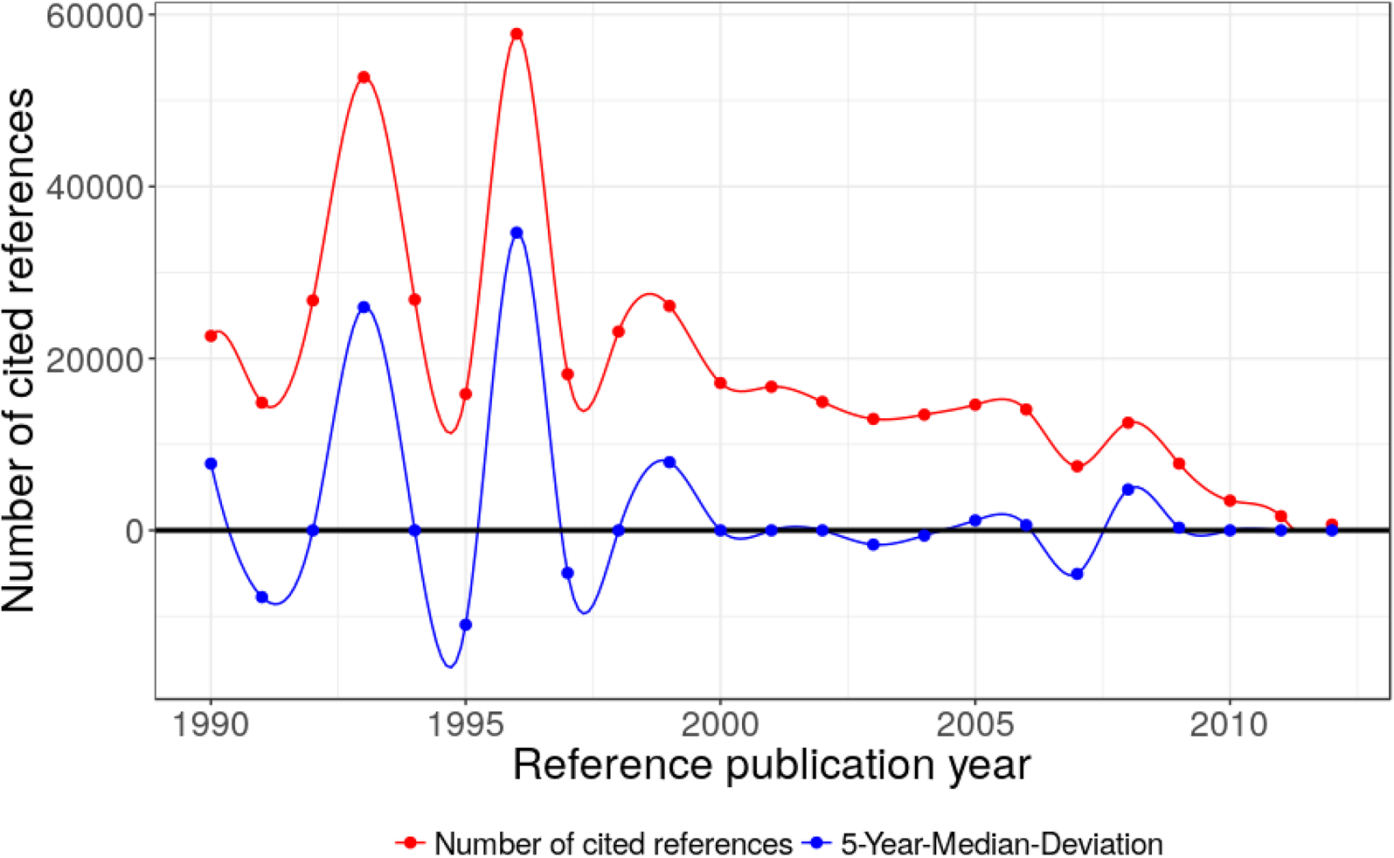
Annual distribution of the references cited in DFT publications across their reference publication years within the time period 1990–2012. The major peak positions are at 1992/1993/1994, 1996, and 1998/1999

In our analysis on the history of DFT from a chemical perspective, we have found several groups of papers which are of high relevance to the DFT research field. Of course, the most important group of papers consists of methodological papers on DFT and its approximations, starting with the famous papers by Hohenberg and Kohn (CR54) and Kohn and Sham (CR55) with a total of 17,847 cited references. This is shown as a peak in 1964/1965 in Fig. 3. The 1980s and 1990s are dominated by several publications of new density functional approximations or improvements to existing ones. The main peaks are 1988 (CR65, CR66) in Fig. 3 (NCR = 38,103), and 1993 (CR69) (NCR = 25,970) as well as 1996 (CR72–CR74) (NCR = 29,522) in Fig. 4. Some of these papers on new approximations and improvements are also dealing with new basis sets as well as tests and comparisons of DFT approximations.

The other group of papers consists of fundamental papers from quantum mechanics and quantum chemistry which are important predecessors of DFT. Although this starts with the foundation of quantum mechanics the most highly cited papers are about approximate methods and their applications. In the spectrogram of the time period 1900 to 1949 we find 6 major peaks in 1928 (3 papers, NCR = 500), 1930 (3 papers, NCR = 812), 1934 (2 papers, NCR = 2733), 1937 (3 papers, NCR = 748), 1944 (1 paper, NCR = 739), and 1947 (1 paper, NCR = 377). However, this spectrogram is clearly dominated by the famous paper of Møller and Plesset (CR37, 1934) on the perturbation theory of many-electron systems (NCR = 2243).

Finally, the third group of papers deals with a broad set of physical and chemical phenomena which have been calculated with various approximations of DFT. Some long-known experiments are revisited in the light of modern quantum chemistry, i.e. DFT. Examples from the 19th century are the Grotthus mechanism for proton transfer in water (CR1), van der Waals theory on capillarity (CR13), the nature of light in magnetic fields (CR4), and the study of several chemical compounds together with their synthesis (CR5–CR12 and CR14). In later periods, the publications on specific physical phenomena and chemical compounds are hidden in the spectrograms since they did not obtain high enough citation rates compared to progresses in theoretical research.

Often, older publications are affected by obliteration by incorporation [74]. This leads to lower citation counts than expected. This is not the case for several publications discussed here, e.g. CR54 and CR55. Kutzelnigg [64] explains the high citation rates of the papers from Hohenberg and Kohn (CR54, 1964) and from Kohn and Sham (CR55, 1965) by mystification of these papers in DFT. Therefore, no obliteration by incorporation occurred. Also, the fact that “DFT” and “Kohn–Sham methods” have been used as synonyms might have led to an unusually high citation count of CR55. This unusually high citation count is not unjustified considering that the vast majority of practical applications employ the Kohn–Sham approach to DFT.

Our study is not without limitations: cited references are included for publications only since 1996 in CAS databases. Therefore, references for earlier publications could not be included in our analysis. Although CAplus has a focus on chemistry, neighboring research areas such as physics and biology are often better covered than one might think. From our experience, CAplus has a broad coverage of the DFT literature (see also a very recent RPYS analysis on the topic of DFT using different databases [75]). Therefore, the major limitation of our study should be missing cited references in publications before 1996. However, RPYS is a rather robust method and the main conclusions should not be affected. Furthermore, the vast majority of DFT publications appeared after 1996 [68].

Very recently, Haunschild and Marx [75] compared co-citation RPYS (RPYS-CO) results using Becke [25] as a marker paper with RPYS results by Haunschild et al. [68] which were based on CAS data. Haunschild and Marx [75] used Web of Science and Microsoft Academic as databases which have cited references for all indexed papers. They found a surprisingly high agreement between RPYS results based on CAS data and RPYS-CO results based on Web of Science data and Microsoft Academic data. This reassures us that the missing cited references before 1996 should not have distorted our results.

We could not mention each frequently cited reference or include it in the tables in “Appendix”. Some seminal papers are concealed by even more cited references in the same or neighboring years. One of such cases is Runge and Gross [63] (the theoretical foundation of TD-DFT) which has an NCR value of 1986 in our RPYS analysis. Despite this rather high NCR value, there is no peak in the spectrogram. In this case, CR62, CR63, and CR64 conceal the paper introducing the theoretical foundations of TD-DFT.

## Data Availability

Our bibliometric study is based on the use of the commercial database CAplus from Chemical Abstracts Service. Although these databases are not available via open access we assume that almost every chemist has access to this database either via SciFinder or via STN. The procedure can also be done using a completely free to access data set from CrossRef (https://search.crossref.org/) as CRExplorer also imports CrossRef downloads in JSON format. Similar results are obtained using the data set deposited at: https://ivs.fkf.mpg.de/dft-rpys/DFT_crossref_download.tgz. However, CrossRef data have many different cited reference variants so that extensive manual merging is necessary. Furthermore, CrossRef does not provide high-quality indexing as CAS does, and both data providers have different coverage of the DFT literature. CAS more exhaustively indexes literature related to chemistry. Therefore, some peak papers observed in RPYS with CAS data do not show up in RPYS analysis using CrossRef data.

## Appendix

See Tables 1, 2, 3 and 4.

**Table 1:** The most frequently cited references from specific reference publication years cited by DTF publications for the time period 1800–1899

N^o^	RPY	Cited reference	NCR
CR1	1806	de Grotthuss, C.J.T. *Mémoire sur la decomposition de l’eau et des corps qu’elle tient en dissolution à l’aide de l’électricité galvanique (Memoir on the decomposition of water and of the bodies that it holds in solution by means of galvanic electricity),* Annales de Chimie 58, 54–74	39
CR2	1819	Petit, A.T.; Dulong, P.L. *Recherches sur quelques points importants de la Théorie de la Chaleur (Research on some important aspects of the theory of heat)*, Annales de Chimie et de Physique 10, 395–413	28
CR3	1825	Faraday, M. *On New Compounds of Carbon and Hydrogen, and on Certain Other Products Obtained during the Decomposition of Oil by Heat*, Philosophical Transactions of the Royal Society of London 115, 440–466	17
CR4	1846	Faraday, M. *XLIX. Experimental researches in electricity.*-*Nineteenth series*, Philosophical Magazine Series 3, 28:187, 294–317, 10.1080/14786444608645086	15
CR5	1850	Strecker, A. *Ueber die künstliche Bildung der Milchsäure und einen neuen, dem Glycocoll homologen Körper (On the artificial formation of lactic acid and a new compound homologous to glycine)*, Justus Liebigs Annalen der Chemie 75, 27–45	7
CR6	1860	Kolbe, H. *Ueber Synthese der Salicylsäure (On the synthesis of salicylic acid)*, Justus Liebigs Annalen der Chemie 113, 125–127	11
CR7	1865	Kekulé, A. *Sur la constitution des substances aromatiques (On the constitution of aromatic substances)*, Bulletin de la Société Chimique 3, 98–111	24
CR8	1869	Glaser, C. *Beiträge zur Kenntnis des Acetenylbenzols (Contributions to the knowledge of acetylene benzene)*, Berichte der deutschen chemischen Gesellschaft 2, 422–424	11
CR9	1874	van‘t Hoff, J.H. *A suggestion looking to the extension into space of the structural formulas at present used in chemistry.* Archives néerlandaises des sciences exactes et naturelles 9, 445–454	25
CR10	1885	Baeyer, A. *Ueber Polyacetylenverbindunge*n *(On polyacetylene compounds)*, Berichte der deutschen chemischen Gesellschaft 18, 2269–2281	14
CR11	1890	Mond, L. *Action of carbon monoxide in Nickel*, Journal of the Chemical Society 57, 749–753	19
CR12	1890	Curtius, T. *Ueber Stickstoffwasserstoffsäure (On hydrazoic acid)*, Chemische Berichte 23, 3023–3033	18
CR13	1894	van der Waals, J. *Thermodynamische Theorie der Kapillarität unter Voraussetzung stetiger Dichteänderung (Thermodynamic theory of capillarity on the condition of continous density change)*, Zeitschrift für Physikalische Chemie 13, 657–724	30
CR14	1894	Fenton, H. *Oxidation of tartaric acid in presence of iron*, Journal of the Chemical Society 65, 899–910	25
CR15	1898	Berthelot, D. *Sur le melange de gaz (On the mixture of gas)*, Comptes Rendus Hebdomadaires des Séances de Académie des Science 126, 1703–1855	8

For each cited reference, a sequential number (N^o^), the corresponding reference publication year (RPY), and the number of cited references (NCR) within the publication set is provided

**Table 2:** The most frequently cited references from specific reference publication years cited by DFT publications for the time period 1900–1949

N^o^	RPY	Cited reference	NCR
CR16	1901	Wulff, G. *Zur Frage der Geschwindigkeit des Wachsthums und der Auflösung der Krystallflächen (On the question of the rate of growth and dissolution of crystal surfaces)*, Zeitschrift für Kristallographie 34, 449–530	107
CR17	1905	Einstein, A. *Motion of suspended particles in stationary liquids required from the molecular kinetic theory of heat*, Annalen der Physik 17, 549–560	33
CR18	1908	Mie, G. *Contributions to the Optics of Turbid Media, Especially Colloidal Metal Solutions*, Annalen der Physik 25, 377–445	41
CR19	1910	Gouy, G. *Sur la constitution de la charge electrique a la surface d’un electrolyte (Constitution of the Electric Charge at the Surface of an Electrolyte)*, Journal de Physique Théorique et Appliquée 9, 457–468	35
CR20	1910	Lindemann, F. *Über die Berechnungen molekularer Eigenfrequenzen (The Calculation of Molecular Vibration Frequencies)*, Physikalische Zeitschrift 11, 609–612	27
CR21	1916	Lewis, G. *The atom and the molecule*, Journal of the American Chemical Society 38, 762–785	49
CR22	1921	Ewald, P. *Die Berechnung optischer und elektrostatischer Gitterpotentiale (The calculation of optical and electrostatic grid potentials)*, Annalen der Physik 64, 253–287	150
CR23	1921	Vegard, L. *Die Konstitution der Mischkristalle und die Raumfüllung der Atom (The constitution of mixed crystals and the space occupied by atoms)*, Zeitschrift für Physik 5, 17–26	89
CR24	1927	Thomas, L. *The calculation of atomic fields*, Proceedings of the Cambridge Philosophical Society 23, 542–548	275
CR25	1927	Fermi, E. *A statistical method for determining some properties of the atom*, Accademia Nazionale dei Lincei 6, 602–507	145
CR26	1927	Born, M.; Oppenheimer, R. *Zur Quantentheorie der Molekeln (Quantum theory of the molecules)*, Annalen der Physik 84, 457–484	101
CR27	1928	Fermi, E. *Eine statistische Methode zur Bestimmung einiger Eigenschaften des Atoms und ihre Anwendung auf die Theorie des periodischen Systems der Elemente (The statistical deduction of some properties of the atom and their application on the theory of the periodic system of elements)*, Zeitschrift für Physik 48, 73–79	233
CR28	1928	Voigt, W. *Lehrbuch der Kristallphysik (Textbook on crystal physics)*, Teubner: Leipzig and Berlin (first published 1910)	172
CR29	1928	Hartree, D., *The wave mechanics of an atom with a non*-*coulomb central field. I. Theory and methods*, Proceedings of the Cambridge Philosophical Society 24, 89–110	95
CR30	1929	Reuss, A., *Berechnung der Fließgrenze von Mischkristallen auf Grund der Plastizitätsbedingung für Einkristalle* (*Calculation of flow limits of mixed crystals on the basis of plasticity of single crystals)*, Zeitschrift fuer Angewandte Mathematik und Mechanik 9, 49–58	207
CR31	1929	Dirac, P., *Quantum mechanics of many*-*electron systems*, Proceedings of the Royal Society of London, Series A: Mathematical, Physical and Engineering Sciences 123, 714–733	196
CR32	1930	Dirac, P., *Exchange phenomena in the Thomas atom*, Proceedings of the Cambridge Philosophical Society 26, 376–385	570
CR33	1930	Fock, V., *Näherungsmethode zur Lösung des quantenmechanischen Mehrkörperproblems (Approximation method for the solution of the quantum mechancial many*-*body problem)*, Zeitschrift fuer Physik 61, 126–148	129
CR34	1930	Eckart, C., *The penetration of a potential barrier by electrons*, Physical Review 35, 1303–1309	113
CR35	1931	Hückel, E., *Quantum*-*theoretical contributions to the benzene problem. I. The electron configuration of benzene and related compounds*, Zeitschrift fuer Physik 70, 204–286	110
CR36	1932	Wigner, E., *Crossing of potential thresholds in chemical reactions*, Zeitschrift fuer physikalische Chemie B 19, 203–216	121
CR37	1934	Møller, C.; Plesset, M., *Note on an approximation treatment for many*-*electron systems*, Physical Review 46, 618–622	2243
CR38	1934	Koopmans, T., *Über die Zuordnung von Wellenfunktionen und Eigenwerten zu den einzelnen Elektronen eines Atoms (On the assignment of wave functions and eigenvalues to the specific electrons of an atom),* Physica 1, 104–113	490
CR39	1935	von Weizsäcker, C., *Zur Theorie der Kernmassen* (*The theory of nuclear masses)*, Zeitschrift fuer Physik 96, 431–458	270
CR40	1935	Eyring, H., *Activated complex in chemical reactions*, Journal of Chemical Physics 3, 107–115	214
CR41	1936	Onsager, L., *Electric moments of molecules in liquids*, Journal of the American Chemical Society 58, 1486–1493	446
CR42	1937	London, F., *Théorie quantique des courants interatomiques dans les combinaisons aromatiques (Quantum theory of interatomic currents in aromatic compounds*), 1937, Journal de Physique et Le Radium 8, 397–409	412
CR43	1937	Hellmann, H., *Einführung in die Quantenchemie (Introduction to quantum chemistry)*, Deuticke: Leipzig und Wien	178
CR44	1937	Jahn, H.; Teller, E., *Stability of Polyatomic Molecules in Degenerate Electronic States. I. Orbital Degeneracy*, Proceedings of the Royal Society of London. Series A, Mathematical and Physical Sciences 161, 220–235	158
CR45	1938	Evans, M.; Polanyi, M., *Inertia and driving force of chemical reactions*, Transactions of the Faraday Society 34, 11–24	153
CR46	1939	Feynman, R., *Forces in molecules*, Physical Review 56, 340–343	304
CR47	1942	Pitzer, K.S.; Gwinn, W.D., *Energy levels and thermodynamic functions for molecules with internal rotation. I. Rigid frame with attached tops*, The Journal of Chemical Physics 10, 428	111
CR48	1944	Murnaghan, F., *The compressibility of media under extreme pressures*, Proceedings of the National Academy of Sciences of the USA (PNAS) 30, 244–247	739
CR49	1945	Herzberg, G., 1945, *Infrared and Raman Spectra of Polyatomic Molecules*, Van Nostrand: New York	173
CR50	1947	Birch, F., *Finite Elastic Strain of Cubic Crystals*, Physical Review 71, 809–824	377

For each cited reference, a sequential number (N^o^), the corresponding reference publication year (RPY), and the number of cited references (NCR) within the publication set is provided

**Table 3:** The most frequently cited references from specific reference publication years cited by DFT publications for the time period 1950–1989

N^o^	RPY	Cited reference	NCR
CR51	1951	Slater J., *A Simplification of the Hartree*–*Fock Method*, Physics Reviews 81, 385–390	737
CR52	1951	Roothaan, C., *New developments in molecular orbital theory*, Reviews of Modern Physics 23, 69–89	381
CR53	1955	Mulliken, R., *Electronic population analysis on LCAO*-*MO molecular wave functions*, Journal of Chemical Physics 23, 1833–1840	1700
CR54	1964	Hohenberg, P.; Kohn, W., *Inhomogeneous electron gas*, Physical Review B 136, 864–871	8213
CR55	1965	Kohn, W.; Sham, L.J., *Self*-*consistent equations including exchange and correlation effects*, Physical Review A 140, 1133–1138	9634
CR56	1970	Boys, S.; Bernardt, F., *The calculation of small molecular interactions by the differences of separate total energies. Some procedures with reduced errors*, Molecular Physics 19, 553–566	3196
CR57	1972	Hehre,W., *Self*-*consistent molecular orbital methods. XII. Further extensions of Gaussian*-*type basis sets for use in molecular orbital studies of organic molecules*, Journal of Chemical Physics 56, 2257–2261	2659
CR58	1973	Hariharan, P., *Influence of polarization functions on MO hydrogenation energies*, Theoretica Chimica Acta 28, 213–222	3001
CR59	1973	Baerends, E., *Self*-*consistent molecular Hartree*–*Fock*-*Slater calculations. I. Computational procedure*, Chemical Physics 2, 41–51	1258
CR60	1976	Monkhorst, H., *Special points for Brillouin*-*zone integrations*, Physical Review B 13, 5188–5192	6506
CR61	1980	Vosko, S., *Accurate spin*-*dependent electron liquid correlation energies for local spin density calculations: a critical analysis*, Canadian Journal of Physics 58, 1200–1211	6046
CR62	1985	Hay, P., Ab initio effective core potentials for molecular calculations—potentials For K to Au including the outermost core orbitals, Journal of Chemical Physics, 82, 299–310	3920
CR63	1985	Hay, P., Ab initio effective core potentials for molecular calculations—potentials for the transition-metal atoms Sc to Hg, Journal of Chemical Physics, 82, 270–283	2995
CR64	1986	Perdew, J., *Density*-*functional approximation for the correlation energy of the inhomogeneous electron gas*, Physical Review B 33, 8822–8824	6106
CR65	1988	Lee, C., *Development of the Colle*-*Salvetti correlation*-*energy formula into a functional of the electron density*, Physical Review B 37, 785–789	23,953
CR66	1988	Becke, A., *Density*-*functional exchange*-*energy approximation with correct asymptotic behavior*, 1988, Physical Review A 38, 3098–3100	14,150

For each cited reference, a sequential number (N^o^), the corresponding reference publication year (RPY), and the number of cited references (NCR) within the publication set is provided

**Table 4:** The most frequently cited references from specific reference publication years cited by DFT publications for the time period 1990–2012

N^o^	RPY	Cited reference	NCR
CR67	1992	Perdew, John; Wang, Yue, *Accurate and simple analytic representation of the electron*–*gas correlation energy*, Physical Review B 45, 13244–13249, 10.1103/PhysRevB.45.13244	5925
CR68	1992	Perdew, John; Chevary, John ALexander; Vosko, Seymour, Jackson, Koblar; Pederson, Mark; Singh, DJ; Fiolhais, Carlos, *Atoms, molecules, solids, and surfaces: Applications of the generalized gradient approximation for exchange and correlation*, Physical Review B 46, 6671–6687, 10.1103/PhysRevB.46.6671	5152
CR69	1993	Becke, Axel, *Density*-*functional thermochemistry. III. The role of exact exchange*, The Journal of Chemical Physics 98, 5648–5652, http://dx.doi.org/10.1063/1.464913	25,970
CR70	1994	Bloechl, PE, *Projector augmented*-*wave method*, Physical Review B 50, 17953–17979, 10.1103/PhysRevB.50.17953	5661
CR71	1994	Stephens, PJ; Devlin, FJ; Chabalowski, CF; Frisch, MJ, *Ab Initio Calculation of Vibrational Absorption and Circular Dichroism Spectra Using Density Functional Force Fields*, The Journal of Physical Chemistry 98, 11623–11627. 10.1021/j100096a001	4394
CR72	1996	Perdew, John; Burke, Kieron; Ernzerhof, Matthias, *Generalized Gradient Approximation Made Simple*, Physical Review Letters 77, 3865–3868, 10.1103/PhysRevLett.77.3865	16,327
CR73	1996	Kresse, G; Furthmüller, J, *Efficient iterative schemes for* ab initio *total*-*energy calculations using a plane*-*wave basis set*, Physical Review B 54, 11169–11186, 10.1103/PhysRevB.54.11169	7796
CR74	1996	Kresse, G; Furthmüller, J, *Efficiency of* ab initio *total energy calculations for metals and semiconductors using a plane*-*wave basis set*, Computational Materials Science 6, 15–50, 10.1016/0927-0256(96)00008-0	5399
CR75	1998	Barone, Vincenzo; Cossi, Maurizio, *Quantum Calculation of Molecular Energies and Energy Gradients in Solution by a Conductor Solvent Model*, Journal of Physical Chemistry A 102, 1995–2001, 10.1021/jp9716997	1477
CR76	1998	Casida, Mark; Jamorski, Christine; Casida, Kim; Salahub, Dennis, *Molecular excitation energies to high*-*lying bound states from time*-*dependent density*-*functional response theory: characterization and correction of the time*-*dependent local density approximation ionization threshold*, Journal of Chemical Physics 108, 4439–4449, 10.1063/1.475855	1403
CR77	1998	Stratmann, Eric; Scuseria, Gustavo; Frisch, Michael, *An efficient implementation of time*-*dependent density*-*functional theory for the calculation of excitation energies of large molecules*, Journal of Chemical Physics 109(19), 8218–8224, 10.1063/1.477483	1319
CR78	1998	Adamo, Carlo; Barone, Vincenzo, *Exchange functionals with improved long*-*range behavior and adiabatic connection methods without adjustable parameters: the mPW and mPW1PW models*, Journal of Chemical Physics 108, 664–675, 10.1063/1.475428	1051
CR79	1999	Kresse, G; Joubert, D, *From ultrasoft pseudopotentials to the projector augmented*-*wave method*, Physical Review B 59, 1758–1775, 10.1103/PhysRevB.59.1758	5583
CR80	1999	Adamo, Carlo; Barone, Vincenzo, *Toward reliable density functional methods without adjustable parameters: the PBE0 model*, Journal of Chemical Physics 110, 6158–6170, 10.1063/1.478522	2134
CR81	2001	Te Velde, G et al., *Chemistry with ADF*, Journal of Computational Chemistry 22, 931–967, 10.1002/jcc.1056	1639
CR82	2005	Tomasi, Jacopo; Mennucci, Benedetta; Cammi, Roberto, *Quantum Mechanical Continuum Solvation Models*, Chemical Reviews 105, 2999–3093, 10.1021/cr9904009	1656
CR83	2005	Weigend, Florian; Ahlrichs, Reinhart, *Balanced basis sets of split valence, triple zeta valence and quadruple zeta valence quality for H to Rn: Design and assessment of accuracy*, Physical Chemistry Chemical Physics 7, 3297–3305, 10.1039/b508541a	1147
CR84	2006	Grimme, Stefan, *Semiempirical GGA*-*type density functional constructed with a long*-*range dispersion correction*, Journal of Computational Chemistry 27, 1787–1799, 10.1002/jcc.20495	2163
CR85	2008	Zhao, Yan; Truhlar, Donald, T*he M06 suite of density functionals for main group thermochemistry, thermochemical kinetics, noncovalent interactions, excited states, and transition elements: two new functionals and systematic testing of four M06*-*class functionals and 12 other functionals*, Theoretical Chemistry Accounts 120, 215–241, 10.1007/s00214-007-0310-x	2020

For each cited reference, a sequential number (N^o^), the corresponding reference publication year (RPY), and the number of cited references (NCR) within the publication set is provided

## Abbreviations

ACS: American Chemical Society
ADF: Amsterdam Density Functional
B3LYP: Becke-3-parameter Lee–Yang–Parr
CAS: Chemical Abstracts Service
COSMO: conductor-like screening model
CR: cited reference
DFT: density functional theory
GGA: generalized gradient approximation
KS DFT: Kohn Sham density functional theory
LAPW: linear augmented-plane-wave
MBPT: many-body perturbation theory
MP2: Møller Plesset perturbation theory of 2nd order
NCR: number of cited references
PAW: projector augmented-wave
PBE: Perdew Burke Ernzerhof
RPA: random phase approximation
RPYS: reference publication year spectroscopy
RPY: reference publication year
STN: Scientific Technical Information Network
TDDFT: time-dependent density functional theory
VASP: Vienna ab initio simulation package
LYP: Lee Yang Parr
